# Association of MMP-9 Haplotypes and TIMP-1 Polymorphism with Spontaneous Deep Intracerebral Hemorrhage in the Taiwan Population

**DOI:** 10.1371/journal.pone.0125397

**Published:** 2015-05-01

**Authors:** Wei-Min Ho, Chiung-Mei Chen, Yun-Shien Lee, Kuo-Hsuan Chang, Huei-Wen Chen, Sien-Tsong Chen, Yi-Chun Chen

**Affiliations:** 1 Department of Neurology, Chang Gung Memorial Hospital, Keelung and College of Medicine, Chang-Gung University, Taoyuan, Taiwan; 2 Department of Neurology, Chang Gung Memorial Hospital Linkou Medical Center and College of Medicine, Chang-Gung University, Taoyuan, Taiwan; 3 Department of Biotechnology, Ming Chuan University, Taoyuan, Taiwan; 4 Genomic Medicine Research Core Laboratory, Chang Gung Memorial Hospital, Taoyuan, Taiwan; St Michael's Hospital, University of Toronto, CANADA

## Abstract

**Background:**

Spontaneous deep intracerebral hemorrhage (SDICH) is a devastating stroke subtype. The causes of SDICH are heterogeneous. Matrix metalloproteinase-9 (MMP-9, Gelantinase B) has been shown to relate to stroke and the development of aneurysm and may increase risks of intracerebral hemorrhage. MMP activities are modulated by their endogenous inhibitors, tissue inhibitors of metalloproteinases (TIMPs). We analyzed the genetic variants of *MMP-9* and *TIMP-1* and SDICH susceptibility.

**Methods:**

Associations were tested by logistic regression or general linear models with adjusting for multiple covariables. Multiplicative terms between genes were applied to detect the interaction effects on SDICH. Permutation testing of 1,000 replicates was performed for empirical estimates.

**Results:**

In the group of ≥65 years old (y/o), we found associations of SDICH with rs3787268 (Odds ratio [OR] = 0.48, 95% confidence interval [CI] 0.27 to 0.86, P = 0.01) and haplotype1 (Hap1) (OR = 0.48, 95% CI 0.26 to 0.86, P = 0.014). For *TIMP1* gene, rs4898 was associated with SDICH in the elder male group (OR = 0.35, 95% CI 0.15 to 0.81, P = 0.015). In contrast, in the younger male group, there were associations of SDICH with rs2250889 (OR = 0.48, 95% CI 0.27 to 0.84, P = 0.01) and Hap3 (OR = 0.61, 95% CI 0.38 to 0.97, P = 0.04). We found significant genetic interaction between *TIMP-1* and *MMP-9* in SDICH susceptibility among younger male subjects (P = 0.004). In subjects carrying rs4898 minor allele, carriers with Hap3 had lower SDICH risk than non-carriers (OR = 0.19, 95% CI 0.07 to 0.51, P = 0.001). In addition, this study showed that when young males were exposed to alcohol, Hap3 was a protective factor of SDICH (OR = 0.06, 95% CI 0.01 to 0.27, P = 0.0002). In contrast, when they were exposed to smoke, Hap2 carriers had increased risk of SDICH (OR = 2.45, 95% CI 1.05 to 5.73, P = 0.04).

**Conclusions:**

This study showed modest to moderate effects of *MMP-9* and *TIMP-1* polymorphisms on SDICH risks with significant age differences. *MMP-9* may interact with alcohol to play a role in the SDICH risk in young men.

## Introduction

Spontaneous intracerebral hemorrhage (ICH) is associated with high morbidity and mortality [1, 2]. About 65% to 80% of spontaneous ICH locates at deep parenchyma structure (SDICH), including the basal ganglia, thalamus, brainstem, and cerebellum. The causes of SDICH are most likely heterogeneous, including environmental and genetic factors. Recently, matrix metalloproteinases (MMPs) pathway has been shown to play multiple roles in remodeling of extracellular matrix (ECM), damage of blood-brain barrier (BBB), and inflammation reactions in spontaneous ICH [3–7]. MMPs are a family of zinc/calcium dependent endopeptidases which function in the degradation of ECM given the ability of splintering matrix integrity. Among MMPs, gelatin-binding MMPs were particularly unique in BBB damage because of their ability to digest type IV and type V collagen. These collagen contents are the essential constituents of vascular basement membrane that is connected with surrounding smooth muscle cells in the vascular endothelium [8–10]. MMP-9 (Gelatinase B) participates not only in collagen integrity but also in interaction involving inflammation [11, 12], reactive oxygen species, and nitric oxide [4, 13]. Several cell types in brain have the capacity to produce MMP-9, including endothelial cells, astrocytes, and microglial cells. Degradation of these collagen tissues is believed to be the beginning step for the breakdown of the vessel integrity, which is responsible for the eventual rupture of the vessel walls [8–10]. Degradation of the vascular ECM by MMP-9 has also been suggested to be a cause for angiogenesis and vascular remodeling [14] and may contribute to the development of unstable aneurismal vasculature and increase the risk of ICH [7, 15]. Immunohistochemistry showed higher levels of total MMP-9, active MMP-9, pro-MMP-9, and tissue inhibitors of metalloproteinases (TIMP)-1 and TIMP-3 in the brain arteriovenous malformations (AVM) specimen than in the control samples [14]. MMP-9 was also discovered in the endothelial and peri-endothelial cell layer and infiltrating neutrophils of brain AVM [14]. The proteolytic effects of MMPs were modulated mainly by TIMPs [4]. Each of the four reported endogenous TIMPs was able to interact with any of the MMPs; however, certain combinations between MMPs and TIMPs have been reported, in which TIMP-1 is the main endogenous inhibitor of MMP-9 [4, 12]. Increased mRNA expression of both MMP-9 and TIMP-1 in cerebral aneurysms was found in animal models [16]. In Chinese populations, whereas no association between MMP-9 gene and ICH susceptibility was found in one study [17], *TIMP-1* variation was associated with ICH in male population in another study [18]. To date, there is no report addressing interactions between MMP-9 and TIMP-1 on SDICH susceptibility.

Given the *a priori* evidence of association between SDICH susceptibility and MMP and TIMP pathway, the purpose of this study is to evaluate whether polymorphisms of *MMP-9* and *TIMP-1* would predispose to SDICH risk in the Taiwan population. To account for the effects of heterogeneity of the ICH mechanisms, this study focused on the phenotype of SDICH.

## Methods

### Subjects

Subjects were recruited from the Department of Neurology, Chang Gung Memorial Hospital (CGMH), Linkou Medical Center. Patients with SDICH were diagnosed based on both clinical presentations and computed tomography and were enrolled if a patient or the legal representative was willing to provide written informed consent. Patients with a traumatic hemorrhage or secondary ICH (brain tumor, vascular anomaly, anticoagulants use, abnormal platelet count, prolonged prothrombin time, and prolonged activated partial thromboplastin time) were excluded. When a SDICH patient had compromised capacity to consent because of alteration of consciousness (Glasgow Coma Scale <15) and mentality caused by neurological or medical conditions, the written informed consent was provided by his/her legal representative based on the laws and regulations of Taiwan. Participants of the control group were recruited from subjects with no history of neurodegenerative diseases, inflammatory diseases, and stroke. This study was approved by the Institutional Review Board of CGMH.

### Clinical information

Anthropometric data and 12-hour fasting blood samples were collected from all participants. Hypertension was diagnosed when blood pressure (BP) repeatedly exceeded 140 mm Hg (systolic) and/or 90 mm Hg (diastolic) or when a subject was taking antihypertensive medication to control hypertension. For SDICH cases without prior diagnosis of hypertension, patients were considered to have hypertension when BP measured after the acute phase of SDICH (2 weeks within the onset) repeatedly >140 mm Hg (systolic) and/or 90 mm Hg (diastolic). Body mass index (BMI) was calculated by weight in kilograms divided by squared height in meters. Diabetes mellitus (DM) was defined based on World Health Organization (WHO) criteria [19]. Alcohol use was defined as drinking ≥210 g per week. Smoking was defined as former (adults who have smoked at least 100 cigarettes in their lifetime, but say they currently do not smoke) or current smoking (adults who have smoked 100 cigarettes in their lifetime and currently smoke cigarettes daily or nondaily) [20].

### Selection of SNP and Genotyping

The cytogenetic location of *MMP-9* was at 20q13.12. In the promoter variant of *MMP-9*, we examined rs3918242 (-1562, C/T) which has been shown to influence expression and a subsequent increase in *MMP-9* transcription [21]. In addition, we selected tagSNPs to identify genetic variation and association to phenotypes without the need of genotyping every SNP in *MMP-9* gene region. The tagSNP is a representative SNP in a region with high linkage disequilibrium (LD) represents a group of SNPs called a haplotype. Based on international HapMap data on NCBI Build 36 assembly for Asian population [22], 3 tagSNPs were selected to tag major haplotypes with criteria of minor allele frequency (MAF) >0.05 and r^2^ value >0.8 by Haploview version 4.2 [23], including rs17576 (A/G, missense variant R279Q, exon6), rs3787268 (A/G, intron), and rs2250889 (C/G, missense variant R574P, exon10). It is worth mentioning that according to dbSNP, there is a wide range of variation in rs17576 genotypes frequency among ethnicities, in which G allele could range from 0.22 (as a minor allele) to 1.0 (as a major allele) [22].

For *TIMP-1* gene (cytogenetic locate at Xp11.23), we selected rs4898 (T/C, synonymous, F124F, exon5) which is a strong tag SNP for population CHB (Han Chinese in Beijing, China) and has been discussed in the prior researches addressing spontaneous ICH [17] and cerebral aneurysm [12].

Blood samples from patients were collected for SNP genotyping and the genomic DNA was isolated from peripheral leukocytes using DNA Extraction kit (Stratagene). The genotype of tagSNP of *MMP-9* and *TIMP-1* gene were determined according to a matrix-assisted laser desorption/ionization time-of-flight (MALDI-TOF)-based mini-sequencing genotyping method as previously described [16] and the primer sets used for PCR amplification and mini-sequencing reaction for each SNP region are listed in Table 1.

**Table 1:** Primers used for amplification and mini-sequencing reaction with MALDI-TOF-based mini-sequencing genotyping method.

MMP-9	rs3918242	F: ATGCCTGGCACATAGTAGGC R: TCGGGCAGGGTCTATATTCA Product Size: 420bp MSP_anti:GAGTAGCTGGTATTATAGGC
MMP-9	rs17576	F: ACCATCCATGGGTCAAAGAA R: GGGCTGAACCTGGTAGACAG Product Size: 296bp MSP: CCCCAGGACTCTACACCC
MMP-9	rs3787268	F: TGAGGTGGGAGGATCTCTTG R: AGGGCGAGGACCTAAAAAGA Product: 275bp MSP: TAGAGGATGTCGCTTAAAAC
MMP-9	rs2250889	F: CTTTCCCTCCTCGCTTTCTC R: AGACGTTTCGTGGGTTATCG Product: 249bp MSP: ACTCGGTCTTTGAGGAGC
TIMP-1	rs4898	F: GAATGGTCCCACTGGAAATG R:TAGCCAGAGGGAGCAAGAAA Product Size: 338bp MSP: CACATCACTACCTGCAGTTT

F: forward primer; R: reverse primer; MSP: Mini-sequencing primer

### Statistical analysis and power estimation

The Pearson’s χ^2^-test or t-test was utilized to compare demographic data between controls and cases; all significance tests were two-tailed. For SNPs, Hardy-Weinberg equilibrium (HWE) with significance level set at 0.05 to control SNP quality. Haplotypes in the subjects were reconstructed by PHASE 2.0 [24] and the haplotypes with frequency <1% were excluded from association analysis.

Association analyses were performed first stratified by genders, and then stratified by age of 65 y/o. Elder population was defined as subjects ≥65 y/o. Multivariable logistic regression was used to analyze the phenotype-genotype associations of SDICH with alleles under dominant genetic models. Covariables included age, sex, hypertension, DM, total cholesterol level, smoking, and alcohol use. To examine interaction effects between genes and between genes and environmental risk factors, the multiplicative term of genotypes and risk factors was included and evaluated in the same model as the interaction term. Permutation testing of 1,000 replicates was performed when the preliminary P-value was <0.05 for empirical estimates as a robust alternative to standard parametric tests. We evaluate the ability of detecting an association between a SNP and SDICH by power calculation implemented in QUANTO version 1.0 [25]. In the present case-control study, at the 5% significance level, we had power greater than 0.8 to identify an association under a dominant genetic model when the per-allele genetic effect was greater than an odds ratio of 1.6 and 1.5 when the MAF > 0.1 and 0.2 respectively. Analyses were performed using SAS software version 9.1.3 (SAS Institute, Cary, NC, USA).

## Results

The characteristics of the study groups are presented in Table 2. A total of 326 SDICH patients and 439 controls were included in this study. Hypertension frequency was significantly higher in patients than in controls. The proportion of smoking and alcohol use were significantly higher in male patients than in controls. The proportion of DM was higher in female patients than in controls. Although age between cases and controls are not statistically different, women with SDICH were older than female controls and the entire male group. Therefore, we stratified the groups by genders and by age of 65 y/o.

**Table 2:** Demographic data in patients with spontaneous deep intracerebral hemorrhage and controls.

	Males (n = 441)			Females (n = 324)		
	SDICH^a^	Controls	P-Value	SDICH^a^	Controls	P-Value
	n = 229	n = 212		n = 97	n = 227	
Age (years)	57.0 ± 12.9	59.3 ± 13.0	0.07	64.2 ± 11.9	62.9 ± 10.9	0.33
Hypertension (%)	90.4	48.1	<0.0001	92.8	51.1	<0.0001
Diabetes mellitus (%)	15.4	14.8	0.86	27.1	13.6	0.002
Alcohol use (%)	41.5	19.8	<0.0001	5.2	1.8	0.09
Smoke (%)	58.5	34.0	<0.0001	5.2	1.8	0.09
Body mass index (kg/m^2^)	25.2 ± 4.1	25.7 ± 3.4	0.21	24.7 ± 3.7	25.3 ±3.8	0.17
Total cholesterol (mg/dL)	170.6 ± 43.6	165.5 ± 56.5	0.29	179.9 ± 48.5	174.8 ±60.6	0.46
Triglyceride (mg/dL)	143.6 ± 94.7	147.1 ± 101.9	0.73	136.0 ± 68.7	144.1 ± 73.0	0.37

Data are expressed as percentage or mean ± SE. Comparisons between controls and SDICH cases were analyzed by χ^2^ test or t-test where appropriate. To convert mg/dL to mmol/L, multiply cholesterol values by 0.02586 and triglycerides by 0.011.

^a^SDICH: spontaneous deep intracerebral hemorrhage.

In SDICH patients, except for alcohol consumption that was more frequent in the young male group than in the elderly (47% versus 25.4%, P = 0.002) (data were not shown in Table 2), there was no other difference between the two age groups regarding alcohol use, smoke, DM, hypertension, and hemorrhage size and location in both genders.

### Genotype frequency and association analysis of controls and patients

Frequency and association of each genotype in SDICH and control subjects were shown in Table 3. The minor allele of rs17576 in our population is A allele, instead of G allele in 1000 Genomes data [26].

**Table 3:** Frequencies and associations of the genotypes in patients with spontaneous deep intracerebral hemorrhage and controls.

	Males			Females			All
	SDICH^a^ (%)	Controls (%)	P-Value, OR^b^ (95% CI^c^)	SDICH^a^ (%)	Controls (%)	P-Value	P-Value, OR(95% CI)
**Age ≥65 y/o**	n = 63	n = 75		n = 50	n = 108		
*MMP9*							
rs3918242 CC/CT+TT	76.2/23.8	85.1/14.9	NS^d^	72.0/28.0	79.3/20.7	NS	NS
rs17576 GG/GA+AA	58.7/41.3	60.9/39.1	NS	56.0/44.0	61.7/38.3	NS	NS
rs3787268 GG/GA+AA	38.1/61.9	25.7/74.3	0.019, 0.35(0.14,0.84)	34.0/66.0	25.9/74.1	NS	0.01, 0.48(0.27,0.86)
rs2250889 CC/CG+GG	64.5/35.5	67.6/32.4	NS	64.0/36.0	68.5/31.5	NS	NS
*TIMP1*							
rs4898 TT/CT+CC	66.1/33.9	45.9/54.1	0.015, 0.35(0.15,0.81)	22/78	27.1/72.9	NS	NS
**Age <65 y/o**	n = 166	n = 137		n = 47	n = 119		
*MMP9*							
rs3918242 CC/CT+TT	78.2/21.8	81.6/18.4	NS	84.4/15.6	76.5/23.5	NS	NS
rs17576 GG/GA+AA	55.8/44.2	48.9/51.1	0.05, 0.6(0.32,1.00)	60.0/40.0	58.8/41.2	NS	NS
rs3787268 GG/GA+AA	33.9/66.1	40.2/59.8	NS	29.8/70.2	35.3/64.7	NS	NS
rs2250889 CC/CG+GG	63.6/36.4	51.5/48.5	0.01, 0.48(0.27,0.84)	61.4/38.6	63.0/37.0	NS	NS
*TIMP1*							
rs4898 TT/CT+CC	58.3/41.7	60.3/39.7	NS	31.9/68.1	33.6/66.4	NS	NS

Analysis was performed by logistic regression under dominant genetic model and adjust for age, sex, hypertension, DM, alcohol drinking, smoking, and total cholesterol level.

^a^SDICH: spontaneous deep intracerebral hemorrhage,

^b^OR: Odds ratio,

^c^CI: confidence interval,

^d^NS: non-significant.

Due to the significantly higher incidence of hypertension in both gender patients and higher proportion of DM in female patients, we first evaluated the susceptibility of SNPs to hypertension and DM. In the younger male group (<65 y/o), we found that *TIMP1* rs4898 minor allele was associated with hypertension risk (OR = 2.16, 95% CI 1.24 to 3.76, P = 0.006) and *MMP9* rs3787268 minor allele had a borderline protection from DM risk (OR = 0.5, 95% CI 0.26 to 0.98, P = 0.04). There was no further association regarding DM and hypertension discovered in the other subgroups.

In the group of ≥65 y/o, logistic regression with adjustment of the covariables showed that rs3787268 was associated with SDICH in the male group (OR = 0.35, 95% CI 0.14 to 0.84, P = 0.019) and in the entire elderly cohort (OR = 0.48, 95% CI 0.27 to 0.86, P = 0.01). For *TIMP1* gene, rs4898 was associated with SDCH in the ≥65 y/o male group (OR = 0.35, 95% CI 0.15 to 0.81, P = 0.015). In contrast, in the younger group (<65 y/o), we found that SDICH was associated with rs17576 (OR = 0.6, 95% CI 0.32 to 1.00, P = 0.05) and rs2250889 (OR = 0.48, 95% CI 0.27 to 0.84, P = 0.01) in male patients. There was no significant association between the SNPs and SDICH in the female group.

Estimated frequency of different haplotypes of *MMP-9* in patients with SDICH and controls were shown in Table 4. There was a single block structure composed of rs17576, rs3787268, and rs2250889. Haplotypes were numbered in the order of their frequency in the subjects. The most common haplotypes was haplotype 1 (Hap1, GAC, Table 4). In subjects ≥65 y/o, Hap1 was associated with a protective effect in men and in combined genders (OR = 0.36, 95% CI 0.15 to 0.88, P = 0.025 and OR = 0.48, 95% CI 0.26 to 0.86, P = 0.014, respectively). In contrast, in subjects <65 y/o, Hap3 was associated with a protective effect in men and in combined genders (OR = 0.47, 95% CI 0.26 to 0.83, P = 0.009 and OR = 0.61, 95% CI 0.38 to 0.97, P = 0.04, respectively) (Table 4).

**Table 4:** Frequencies and associations of the *MMP-9* haplotypes carrier in patients with spontaneous deep intracerebral hemorrhage and controls.

	Males			Females			All
	SDICH^a^	Controls	P-Value, OR^b^ (95% CI^c^)	SDICH^a^	Controls	P-Value, OR (95% CI)	P-Value, OR (95% CI)
Age ≥65 y/o	n = 63	n = 75		n = 50	n = 108		
Hap1 carrier	61.9%	72.6%	0.025, 0.36 (0.15,0.88)	65.3%	74.1%	NS^d^	0.014, 0.48 (0.26,0.86)
Hap2 carrier	58.7%	54.8%	NS	57.1%	53.7%	NS	NS
Hap3 carrier	34.9%	30.1%	NS	34.7%	31.5%	NS	NS
Hap4 carrier	7.9%	8.2%	NS	14.3%	7.4%	NS	NS
Age <65 y/o	n = 166	n = 137		n = 47	n = 119		
Hap1 carrier	65.6%	60.3%	NS	70.2%	64.7%	NS	NS
Hap2 carrier	57.1%	58.1%	NS	61.7%	55.5%	NS	NS
Hap3 carrier	36.2%	48.5%	0.009, 0.47 (0.26,0.83)	36.2%	37.0%	NS	0.04, 0.61 (0.38,0.97)
Hap4 carrier	8.6%	5.2%	NS	2.1%	7.6%	NS	NS

Haplotype 1 (Hap1) carrying one or two copies of GAC, Hap2: GGC, Hap3: AGG, Hap4: AGC.

Analysis was performed by logistic regression model and adjust with age, sex, DM, HTN, Alcohol drinking, smoking, and total cholesterol level are adjusted.

^a^SDICH: spontaneous deep intracerebral hemorrhage,

^b^OR: Odds ratio,

^c^CI: confidence interval,

^d^NS: non-significant.

**Fig 1:**
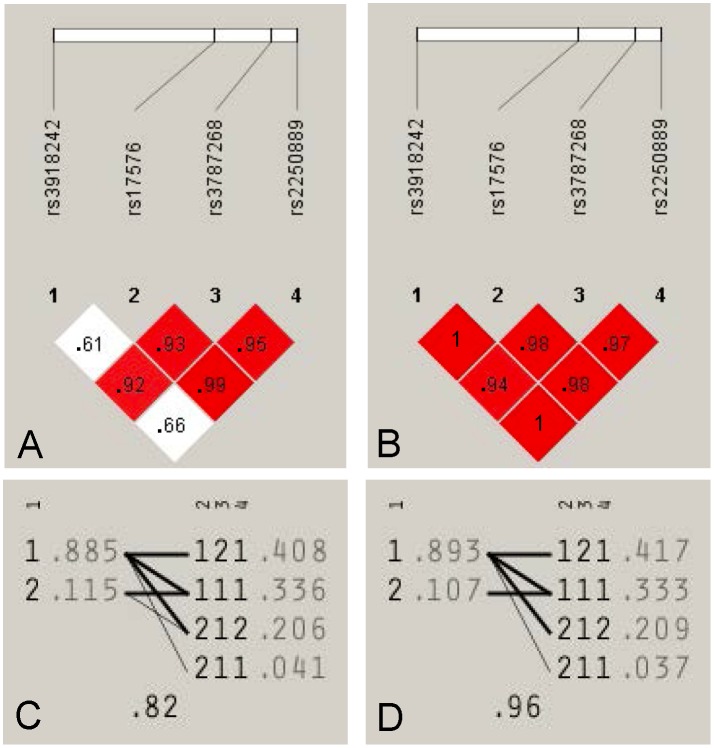
Haploview disequilibrium coefficients (D’) of the pairwise loci in the Intracerebral hemorrhage group and control group. Four SNPs in the genomic region of *MMP-9* loci were analyzed by Haploview version 4.2 software. The D’ value of the pairwise loci were measured from the SDICH group and control group and are shown in Panel A and B, respectively. A D’ value of “1” indicates that the examined two loci exhibit complete linkage while a value of “0” demonstrates the independence of one another. While rs3918242 was weak linkage disequilibrium (LD) with the other 3 SNPs in the SDICH group (Panel A), the four polymorphisms showed strong LD with each other in control group (Panel B). In both group, strong LD was observed between rs17576, rs3787268, and rs2250889. Panel C and D represented levels of recombination between rs3918242 and the block in the SDICH group and control group, respectively. Alleles are displayed using 1 as major allele and 2 as minor allele of each SNPs. Frequencies are shown next to each haplotype and lines show the most common crossings from rs3918242 and the block, with thicker lines showing more common crossings than thinner lines. Shown beneath the crossing lines is multilocus D', which is a measure of the LD between rs3918242 and the block. The amount of historical recombination between the two blocks in SDICH (0.82) was greater than that in controls (0.96).

Haploview disequilibrium coefficients (D’) of the pairwise loci were measured from each of the ICH group and control group (Fig 1). Haploview analysis showed that LD between rs3918242 and rs17576 was different between the SDICH group and controls. While rs3918242 was not in LD in the ICH group, all four SNPs showed strong LD with each other in the control group, representing that level of recombination between rs3918242 and the block was different between the SDICH and control groups. Multilocus D' measured that the amount of historical recombination, and the closer to zero the value is, the greater the amount of historical recombination between rs3918242 and the block. The multilocus D' value indicated that the historical recombination in ICH (0.82) might be greater than that in controls (0.96).

**Fig 2:**
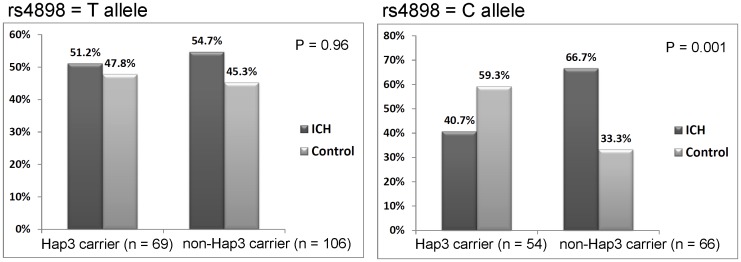
Interaction between *TIMP-1* rs4898 and *MMP-9* haplotype 3 (Hap3) and SDICH susceptibility. Interaction between *TIMP-1* rs4898 and *MMP-9* haplotypes was significant (P = 0.009) in ICH susceptibility among younger male subjects. In subjects carrying rs4898 major allele (T), SDICH risk was similar between Hap 3 carriers and non-carriers (P = 0.96). However, in subjects carrying rs4898 minor allele (C), Hap3 carriers had a significant protective effect from SDICH risk compared to non-Hap3 carriers (OR = 0.21, 95% CI 0.08 to 0.54, P = 0.001). Therefore, rs4898 minor allele and Hap3 had a significant additive protective effect from SDICH susceptibility.

When further considering interaction between genes on SDICH susceptibility, multiplicative terms of *TIMP-1* rs4898 and the *MMP-9* haplotypes evaluated in a similar model showed significant genetic interaction between *TIMP-1* and *MMP-9* (P = 0.004) in SDICH susceptibility among younger male subjects (Fig 2). Specifically, in younger subjects carrying rs4898 major allele (T), SDICH risk was similar between Hap3 carriers and non-carriers (P = 0.96). However, in subjects carrying rs4898 minor allele (C), carriers with Hap3 had a significant protective effect from SDICH risk than non-Hap3 carriers (OR = 0.21, 95% CI 0.08 to 0.54, P = 0.001). We did not find interaction between *TIMP-1* rs4898 and the *MMP-9* haplotypes in the other subgroups.

**Fig 3:**
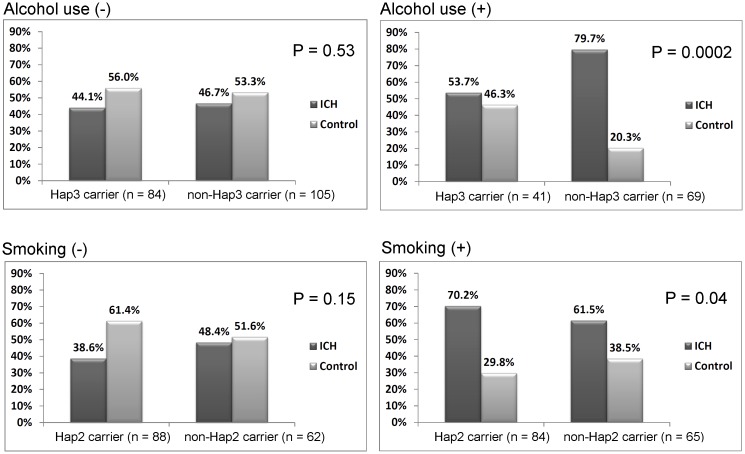
Interaction between *MMP-9* haplotypes and environmental factors to SDICH susceptibility. Interaction between *MMP-9* haplotypes and alcohol consumption was significant (P = 0.004). Specifically, in alcohol-free subjects, SDICH risk was similar between carriers and non-carriers of haplotype 3 (Hap3, P = 0.53), whereas in subjects with alcohol consumption, Hap3 carriers had a significant protective effect than non-Hap3 carriers (OR = 0.06, 95% CI 0.01 to 0.27, P = 0.0002). Interaction between *MMP-9* haplotype 2 (Hap2) and smoke was also significant (P = 0.007). In subjects with smoke, Hap2 carrier had a significant SDICH risk than non-Hap2 carriers (OR = 2.45, 95% CI 1.05 to 5.73, P = 0.04), while in smoke-free subjects, SDICH risk was similar between Hap2 carriers and non-carriers (P = 0.15).

To examine gene-environment interaction, we performed haplotype analyses in all the subgroups and found significant interactions between Hap3 and alcohol consumption as well as Hap2 and smoke in the younger male group (P = 0.004 and 0.007, respectively). Specifically, SDICH risk was similar between alcohol-free subjects carrying and not carrying Hap3 (P = 0.53), whereas in subjects with alcohol consumption, Hap3 carriers had a significant protective effect than non-Hap3 carriers (OR = 0.06, 95% CI 0.01 to 0.27, P = 0.0002). In contrast, in subjects consuming cigarette, Hap2 carrier had a significant SDICH risk than non-Hap2 carriers (OR = 2.45, 95% CI 1.05 to 5.73, P = 0.04), while risk was similar between smoke-free subjects carrying and not carrying Hap2 (P = 0.15) (Fig 3). There was no interaction between alcohol consumption and rs3918242 as well as rs4898.

## Discussion

The study herein is the first showing that the common genetic variations of *MMP-9* and *TIMP-1* genes were associated with SDICH susceptibility with significant age differences. In the elderly group, we found that carriers of minor allele A of rs3787268 tended to be protected from SDICH. Although association analysis of rs3787268 in the female group did not reach statistical significance, there was a same protection trend in females. On the other hand, in male subjects younger than 65 years old, carriers of minor alleles of rs17576 and rs2250889 tended to be protected from SDICH as well. The protective effects of the variants rs17576 and rs2250889 are most likely independent of hypertension pathway and DM, because the two variants were not related to hypertension and DM. Although in the younger male group, *TIMP1* rs4898 variant was associated with hypertension risk and *MMP9* rs3787268 variant had a borderline protection from DM risk, none of them were associated with SDICH risks in the young males. We also found that when young males exposed to alcohol and smoke, Hap3 was a protective factor and Hap2 carriers had increased risk to SDICH, respectively. These findings suggested that carrying the minor alleles of these tagSNPs is protective from SDICH risks. Although hypertension is the most important risk factor of young SDICH [27] and could contribute to the familial aggregation of SDICH, it accounted for 54% of SDICH cases [28], and additional effects of susceptibility genes of novel pathophysiology were suggested in the previous studies [29, 30]. We previously reported that male subjects carrying genotype *APOE* ε2ε3 tend to have a higher SDICH risk than ε3ε3 carriers when they have alcohol exposure, but may have more benefit from alcohol abstinence [31]. The report herein suggests that *MMP-9* may interact with alcohol to play a role in the SDICH risk in the younger male group. Interestingly, while haplotype analyses indeed provided higher power to detect an association, there was discrepancy of the associations between the elder and the younger group. In the elderly group, Hap1 was a protective factor whereas, in the younger group, Hap3 was a protective factor of SDICH, suggesting possible different pathophysiology of SDICH in different age groups. It is worth mentioning that although there was gender difference in significance of the Hap1 and Hap3 associations with SDICH, the trend of haplotype distributions in SDICH and controls was similar between genders. Further replication study with large sample size is rendered to confirm this association. Hap3 was composed of minor alleles of rs17576 and rs2250889. Missense variation of rs17576 led to R279Q in exon 6 of the catalytic domain of the MMP-9 enzyme, causing alterations in protein binding site and charged affinity [22]. Similarly, missense variation of rs2250889 leading to R574P in exon 10 of pexin-like domain might also cause changes in MMP-9 polarity, structure, and function given the substantial replacement of arginine. Therefore, we assume that activity of MMP-9 may be changed by the genetic variations rs17576 and rs2250889, which warrants further functional studies to clarify. This study did not discover association between the promoter SNP rs3918242 and SDICH risk. In summary, this study suggested that *MMP-9* genetic variations may influence SDICH susceptibility with age difference.

Age difference in the role of MMPs and TIMPs pathway on cerebral aneurysms has been addressed in a prior animal model [16], in which quantitative PCR showed an increasing of TIMP-1 mRNA in the early stage of aneurysm progression but not in the late stage, whereas mRNA expression of MMP-9 increased in the late stage [16]. Generally, the increase in MMP-9 activity may be a risk of cerebral vascular abnormality [32] and TIMP-1 has a protective role for aneurysm progression [16]. However, MMP-9 can function dual roles on neuroprotection and angiogenesis [5] and the compensation of MMP-2 and MMP-3 expression in response to low MMP-9 deficiency knock-out mice has been demonstrated [33], suggesting a complexity of the MMPs-TIMPs pathway. In our genetic approach, we found that C allele of rs4898 indeed provides a protective effect on SDICH risk in the elderly male group, but not in the young [34]. Age and gender difference should be considered in any future study addressing the MMPs-TIMPs pathway. A prior study [17] showed that variation of *TIMP-1* rs2070584 but not rs4898 was associated with ICH in the male population. However, age stratification was not performed in their report. Nevertheless, they demonstrated higher serum levels of TIMP-1 in ICH patients than in controls, even though no correlation between TIMP-1 levels and genotypes was found in their study. Furthermore, in our subjects <65 y/o, carriers of rs4898 minor allele and Hap3 (minor haplotypes of *MMP-9*) had lower SDICH risk than non-carrier, suggesting a gene-gene interaction of protective effect of these genetic variations. Therefore, rs4898 minor allele and Hap3 may have a significant additive protective effect from SDICH susceptibility [35].

This study included a homogenous disease entity in a same ethnic background, which may limit the confounding effect from multiple phenotypes and ethnicities. This is the first study proposing *MMP-9* and *TIMP-1* genotypes and their interactions to the SDICH susceptibility with age difference. However, because the number of female cases is relatively small, genetic effect that was less than 1.5 may not be identified in this study. Further replicated study is needed to confirm the results herein, especially for the female population to avoid a false negative result. In the present study, female patients with SDICH were older than female controls and the entire male group. Our group previously reported that female gender may be a protective factor of SDICH [27]. Estrogen was related to nitric oxide production [36] and nuclear factor kappa B pathway, which may be influenced by MMP-9 [37]. In addition, protective effect of estrogen on intracranial aneurysm rupture has been shown in animal model [38]. Future study addressing the interaction between estrogen effect and *MMP-9* and *TIMP-1* in the SDICH risk is needed. It is worth mentioning that the SNPs we studied may not pose a specific risk factor for SDICH, given that these SNPs could be a risk factor for numerous conditions. Although the effect sizes are moderate in certain subgroups, the study needs to be replicated before these SNPs can be viewed as independent risk factors for SDICH. Another limitation of this study is that we did not measure serum levels of MMP-9 and TIMP-1, which may otherwise provide additional functional information to support our hypothesis. Also, except for anticoagulants, we did not record information of medications, which may also be a study limitation.

## Conclusions

This study showed a modest to moderate effects of *MMP-9* and *TIMP-1* polymorphisms on SDICH risks with significant age differences. There was interaction between *MMP-9* haplotypes and *TIMP-1* polymorphisms on SDICH susceptibility in male subjects younger than 65 years old. When young males exposed to alcohol, Hap3 was a protective factor of SDICH. In contrast, when young males exposed to smoke, Hap2 carriers had increased risk of SDICH.

## Supporting Information

S1 File The data underlying the findings in our study are available in the supplemental file. (XLSX)
